# Hwa-byung (anger syndrome) as a risk factor for suicidal ideation in MZ generation: a survey study in South Korea

**DOI:** 10.3389/fpsyg.2024.1461750

**Published:** 2024-12-09

**Authors:** Chan-Young Kwon

**Affiliations:** Department of Oriental Neuropsychiatry, College of Korean Medicine, Dong-Eui University, Busan, Republic of Korea

**Keywords:** suicidal ideation, generation MZ, hwa-byung, depression, anger

## Abstract

**Introduction:**

Suicide is a critical global public health issue, with South Korea exhibiting the highest suicide rate among OECD countries at 24.1 per 100,000 people in 2020. This study focuses on suicidal ideation (SI) within South Korea’s Millennials and Generation Z (Generation MZ) by examining the impact of anger and hwa-byung (HB), a culture-bound anger syndrome.

**Methods:**

The online survey was conducted between June 7 and 12, 2024. The inclusion criteria for this study were the following: (1) generation MZ (i.e., those born between 1980 and 2005); (2) those without a history of mood disorders (i.e., depressive disorders or bipolar disorder); (3) those of Korean nationality and residing in South Korea. Demographic variables and clinical variables including SI, HB, and depression were investigated. The chi-square test or t-test was used to compare the differences between the SI and non-SI groups. Additionally, binary logistic regression analysis was performed to analyze factors associated with the presence of SI. Finally, Pearson’s correlation coefficients were calculated to explore HB symptoms that were highly associated with SI.

**Results:**

Total 457 participants were included. The survey revealed an overall prevalence of SI of 38.07%, with 18.82% reporting “much” or more SI and 4.81% reporting “very much” SI. Significant differences were found between the SI and non-SI groups in HB symptoms, depression, anxiety, perceived stress, trait anger, state anger, anger-in, and anger-out (all *p* < 0.001). Logistic regression analysis identified HB symptoms (odds ratio [OR], 1.05; 95% CI, 1.00–1.11; *p* = 0.050), depression (OR, 1.41; 95% CI, 1.23–1.62; *p* < 0.001), and state anger (OR, 1.14; 95% CI, 1.05–1.24; *p* = 0.002) as significant factors for SI. The mediating factors confirmed the direct and indirect effects of HB symptoms on the presence of SI. Pearson’s correlation coefficients between HB symptoms and SI severity ranged from 0.241 to 0.536, with physical symptoms, such as heat sensation and chest pressure, showing high correlations (0.426 to 0.476).

**Conclusion:**

These findings highlight the need for mental health policies that integrate Korean medical approaches into suicide prevention. Future research should confirm these results using larger, nationally representative samples to improve generalizability and further explore HB and suicidality in diverse populations.

## 1 Introduction

Suicide is an urgent public mental health concern worldwide (Henry, 2021). Among them, South Korea, in particular, has a high suicide rate worldwide and the highest suicide rate among the Organization for Economic Cooperation and Development (OECD) countries, with a suicide rate of 24.1 per 100,000 people in 2020 (OECD, 2024). Suicidal ideation (SI), one of the suicidal behaviors, is a major target for suicide prevention (Mann et al., 2021). SI exists on a spectrum ranging from a general desire for death to a firm’s intention to act on that thought actively (Harmer et al., 2024). Factors known to be significantly associated with the presence of SI in Koreans differed depending on age group, but depression and perceived stress were both significant risk factors in all age groups (Hwang and Park, 2021).

Generation MZ, which stands for “Millennials” and “Generation Z,” has become Korea’s main working-age group. This generation values the agreement between individual and organizational values and wants the meaning of what individuals pursue to be respected (Moon and Kim, 2023). This generation shows distinct characteristics, particularly their heightened sensitivity to fairness and justice (Moon et al., 2024). In this generation, unfairness and injustice play an important role in turnover intention (Moon et al., 2024) and mental health outcomes (Kim, 2020). Considering that higher perceived overall injustice is associated with higher levels of anger (Qin and Zhang, 2022), the emotion of anger among generation MZ in South Korea is likely to have a significant impact on their life (Hong and Hong, 2023). This generation’s unique characteristic of sensitivity to injustice makes them particularly vulnerable to hwa-byung (HB), a Korean culture-bound anger syndrome specifically associated with perceived unfairness and suppressed anger (Lee et al., 2014; Kwon et al., 2020). It is characterized by the suppression of anger caused by perceiving unfair or unjust social experiences, leading to both psychological and physical symptoms (Min, 2008; Kwon et al., 2020). Min validated the diagnostic criteria of HB through factor analysis, identifying core symptoms including feelings of unfairness, subjective anger, expression of anger, heat sensation, and respiratory discomfort (Min et al., 2009). The prevalence of HB in the Korean general population has been reported to be approximately 4.2 to 13.3%, and it is more commonly diagnosed in individuals experiencing chronic stress and perceived injustice (The Korean Society Of Oriental Neuropsychiatry, 2021).

Korean medicine, a traditional medical system that has been officially recognized alongside Western medicine in South Korea’s healthcare system (Park et al., 2021), plays a primary role in diagnosing and treating HB (Kwon and Lee, 2024). Korean medicine conceptualizes HB within its unique theoretical framework that emphasizes the mind–body connection and the pathogenic role of emotional disturbance (Lee et al., 2014). In the Korean medical system, patients with HB are primarily diagnosed and treated in Korean medicine clinics, where practitioners employ diagnostic methods including pattern identification and traditional symptom assessment (Kwon and Lee, 2024). This cultural and medical context is particularly important as Korean medicine doctors are often the first healthcare providers to identify and manage HB symptoms, though they are currently not included in South Korea’s national suicide prevention policies (Kwon and Lee, 2024).

Emotional labor, defined as a means for employees to manage their emotions and to express only those requested by their organizations (Hochschild, 2019), encompasses surface acting (modifying external expressions) and deep acting (modifying both feelings and expressions) (Ashforth and Humphrey, 1993). This concept is particularly relevant to the MZ generation in South Korea, who predominantly work in service-oriented industries requiring high levels of emotional regulation (Kim S. S. et al., 2023; Lee et al., 2023). Given that emotional labor often involves suppressing negative emotions, especially anger, to maintain appropriate workplace behavior (Kim et al., 2019), it may share psychological mechanisms with HB, where anger is typically suppressed due to social and cultural constraints (Lee et al., 2014; Kwon et al., 2020). Additionally, in this context, high emotional demands associated with emotional labor were found to be related to the presence of SI in service and sales workers in South Korea (Yoon et al., 2016).

While anger has been studied in the context of suicide risk and was found to be significantly related to SI and/or suicidal behavior in studies with Koreans (Lee et al., 2009; Bagalkot et al., 2014), the impact of culturally specific anger expression, particularly HB, on SI among generation MZ remains understudied. This gap is particularly concerning given that this generation’s characteristic sensitivity to injustice may manifest differently in terms of anger expression and related mental health outcomes compared to other generations (Kim, 2020; Moon et al., 2024). In South Korea, which has the highest suicide rate among OECD countries (OECD, 2024), identifying SI-related risk factors across generations will contribute to improving suicide prevention strategies.

Therefore, this study sought to analyze the contribution of HB, a unique cultural manifestation of anger related to perceived injustice, to SI in generation MZ, aiming to inform culturally appropriate suicide prevention strategies for this population.

## 2 Methods

### 2.1 Study design

This study was carried out using an anonymous online survey. The online survey was conducted between June 7 and 12, 2024 by Macromill Embrain (Embrain Co., Ltd., Seoul, Korea).

### 2.2 Participants

According to the Korean government, as of 2020, the number of generation MZ was 16.299 million, accounting for 32.5% of the total population (KOCIS, 2024). A total of 54.9% of Generation M and 50.2% of Generation Z lived in metropolitan areas (i.e., Seoul, Gyeonggi-do, and Incheon). Considering the population size of the subjects and a 5% error margin with a 95% confidence level, the target number of subjects for this survey was calculated to be 384 (Serdar et al., 2021). Therefore, we aimed to recruit at least 400 participants to account for potential incomplete responses. The inclusion criteria for this study were the following: (1) generation MZ (i.e., those born between 1980 and 2005); (2) those without a history of mood disorders (i.e., depressive disorders or bipolar disorder); (3) those of Korean nationality and residing in South Korea. The survey link was sent to the company panel.

### 2.3 Variables

#### 2.3.1 Demographic variables

Demographic variables, including sex, age, area of residence, and marital status, were investigated.

#### 2.3.2 Clinical variables

##### 2.3.2.1 SI

The presence of SI was assessed with the question “Within the past week I had thoughts about taking my own life.” Participants responded to this question with “not at all,” “a little,” “much,” and “very much.” All participants who did not respond “not at all” were considered to have SI.

##### 2.3.2.2 HB

The HB scale was used to evaluate HB traits and HB symptoms. This scale consisted of 16 questions evaluating HB traits and 15 questions evaluating HB symptoms (Kwon et al., 2008). Respondents responded to each question on a 0-4-point Likert scale. The items that evaluated the symptoms of HB included psychological and physical symptoms. In particular, the 15th question on HB symptoms is “I think the world is unfair,” which assesses the perceived injustice of the respondents. In this study, the Cronbach’s alpha coefficient calculated in the current study were 0.88 for HB traits and 0.94 for HB symptoms, respectively (0.85 and 0.83, respectively, in the original study).

##### 2.3.2.3 Emotional labor

Emotional labor was assessed using Lee’s emotional labor scale (Lee, 2007), which was developed based on Grandey (2000) conceptual model of emotional labor. The scale consists of 14 items evaluating two dimensions: employee-focused emotional labor (6 items assessing surface acting and deep acting) and job-focused emotional labor (8 items assessing frequency, duration, and variety of emotional expressions). Each item is rated on a 5-point Likert scale, with higher scores indicating higher levels of emotional labor. High emotional demand was included in this survey because it could potentially be a risk factor for SI among Koreans (Yoon et al., 2016). In this study, the Cronbach’s alpha coefficient calculated in the current study were 0.81 for employee-focused emotional labor and 0.86 for job-focused emotional labor, respectively (0.78 to 0.80 in the original study).

##### 2.3.2.4 Depression, anxiety, and perceived stress

The Depression, Anxiety, and Stress Scale-21 (DASS-21), which consists of 21 questions, was used. Depression, anxiety, and stress were each assessed using seven questions and all questions were answered on a 0–3 point Likert scale (Ng et al., 2007). In this study, the Cronbach’s alpha coefficient calculated in the current study were 0.92 for depression, 0.90 for anxiety, and 0.89 for stress, respectively [0.93 in a recent Korean study (Hwang et al., 2024)].

##### 2.3.2.5 Anger

The State–Trait Anger Expression Inventory was used to assess trait anger, state anger, anger-in, anger-out, and anger-control (Spielberger et al., 2013). Respondents answered 44 questions on a 1–4 point Likert scale. In this study, the Cronbach’s alpha coefficient calculated in the current study were 0.91 for trait anger, 0.95 for state anger, 0.87 for anger-in, 0.88 for anger-out, and 0.83 for anger-control, respectively [0.71 to 0.96 in a recent Korean study (Lee et al., 2024)].

##### 2.3.2.6 Subjective health status

Respondents responded with their subjective perception of their health status as “very bad,” “bad,” “average,” “good,” and “very good.”

##### 2.3.2.7 Chronic diseases

The presence of chronic diseases was determined using the form surveyed by the Korea Health Panel Survey, a nationally representative panel in South Korea (Park and Kim, 2022). The participants responded “yes” or “no” to the presence of the following chronic diseases: hypertension, diabetes, liver disease (hepatitis, cirrhosis, etc.), kidney disease (renal failure, etc.), digestive disease (functional dyspepsia, etc.), cardiovascular disease (angina, myocardial infarction, etc.), cerebrovascular disease (cerebral hemorrhage, cerebral infarction, etc.), chronic lower respiratory tract diseases (asthma, emphysema, chronic obstructive pulmonary disease, bronchiectasis, etc.), joint diseases (knee arthrosis, intervertebral disc disorders, other spinal diseases, etc.), hypothyroidism, hyperthyroidism, cancer (stomach cancer, colon cancer, lung cancer, etc.), depressive disorder, bipolar disorder, and dementia. As described, participants with mood disorders including depressive disorder and bipolar disorder were excluded from the analysis.

### 2.4 Data analysis

The demographic information of the participants was analyzed using descriptive statistics. The normality of each variable was assessed using the Kolmogorov–Smirnov test. While the Kolmogorov–Smirnov test suggested deviations from normality (*p* < 0.05), we conducted additional assessments of distribution characteristics. The skewness (< 2) and kurtosis (< 7) values fell within acceptable ranges recommended by Kim (2013). Kim (2013), indicating that the violations of normality were not severe enough to preclude the use of parametric tests. Therefore, parametric tests such as *t*-tests and Pearson’s correlation coefficients were deemed appropriate for this analysis.

Additionally, multivariable logistic regression analysis was performed to analyze factors associated with the presence of SI. All variables were simultaneously entered into the model to adjust for potential confounding effects. The calculated values were expressed as adjusted odds ratios (aORs) and their 95% confidence intervals (CIs). Three separate multivariable models were constructed for different SI severity thresholds (“a little” or more, “much” or more, and “very much”). Each model included demographic variables (sex, age, marriage status, residence area), clinical variables (subjective health status, chronic disease presence), and psychological variables (HB symptoms, HB traits, emotional labor, depression, anxiety, stress, and anger-related variables) as independent variables, with all variables mutually adjusted for each other.

To examine potential mediating effects of psychosocial factors on the relationship between HB symptoms and SI, a mediation analysis was conducted with the PROCESS macro (Hayes and Rockwood, 2017). Clinical variables that showed significant associations with SI in regression analysis were tested as potential mediators. Demographic variables and clinical variables that were not significantly related were considered covariates. Bootstrap sampling (5,000 samples) was used to estimate the 95% CIs for the indirect effects. Finally, Pearson’s correlation coefficients were calculated to explore HB symptoms that were highly associated with SI.

The analysis of SI was approached in two ways to serve different research objectives. For the logistic regression analysis, SI was dichotomized (presence/absence) at different thresholds to identify clinically relevant risk factors for varying severity levels of SI. This approach aligns with clinical practice where the presence of SI, regardless of severity, warrants attention. For the correlation analysis with HB symptoms, we utilized SI as an ordinal variable to capture the full range of severity and its relationship with specific symptoms, allowing for more nuanced understanding of symptom-level associations.

All statistical analyses were performed with PASW Statistics for Windows (version 18.0; SPSS Inc., Chicago, IL, USA), and *p*-values < 0.05 were considered statistically significant.

### 2.5 Ethical considerations

All participants voluntarily agreed to participate in the survey and consented to the use of their personal information. This study was approved by the Institutional Review Board of Dong-eui University Korean Medicine Hospital (DH-2024-05, approved on May 28, 2024).

## 3 Results

### 3.1 Baseline characteristics of the participants

**Figure 1:**
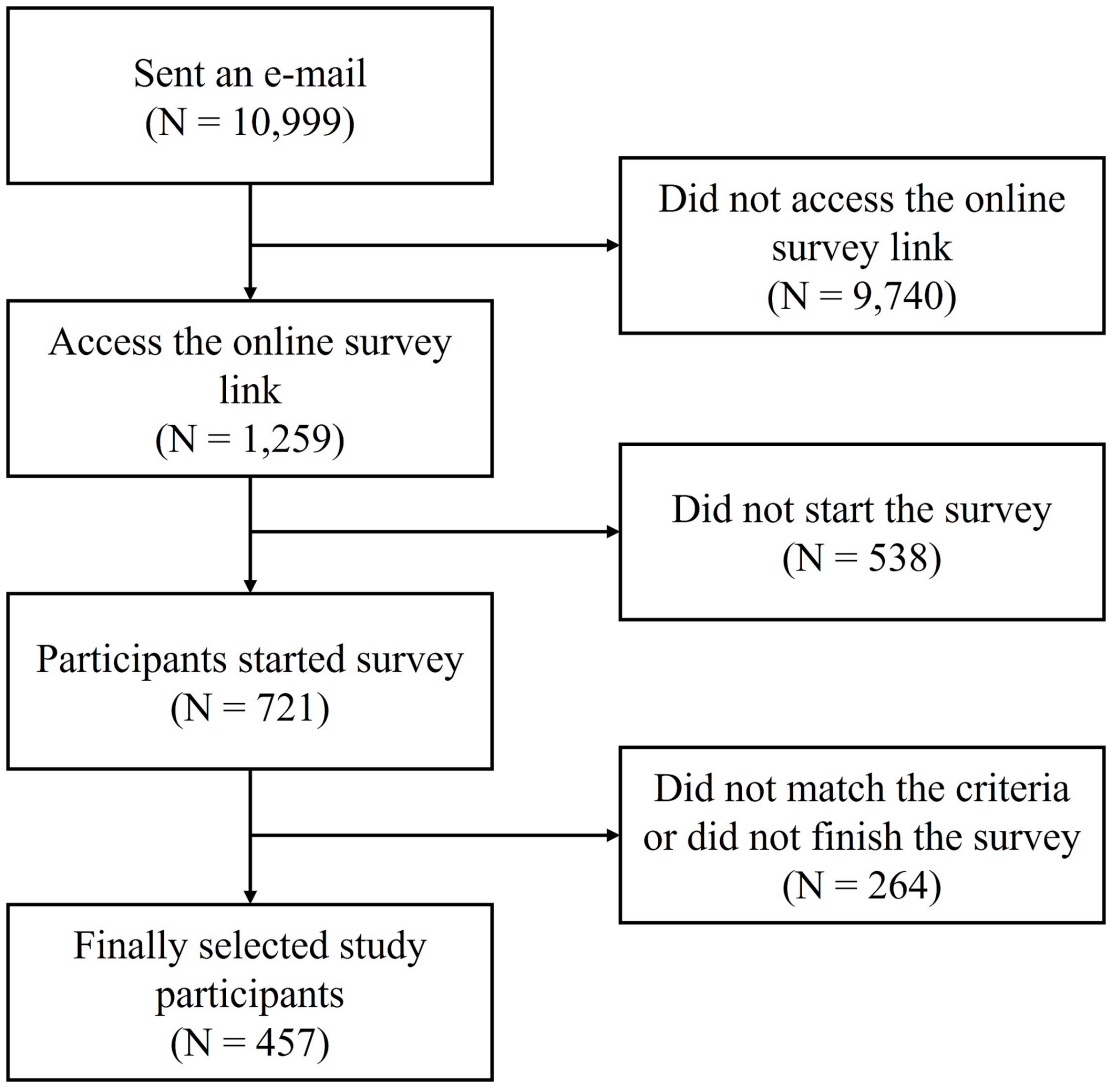
Flow diagram of selection of the study participants.

A total of 457 participants met the inclusion criteria (Figure 1). Among the participants, 214 were women (46.83%), and the average age was 34.75 ± 7.45 years. Among the participants, the number of participants who responded “a little,” “much,” and “very much” to SI was 174, 86, and 22, respectively, and the overall prevalence of SI among these participants was 38.07%. Furthermore, the prevalence rates of “much” or more SI and “very much” SI were 18.82 and 4.81%, respectively. No statistically significant differences were found in sex, age, area of residence, presence of chronic disease, or marital status between the non-SI and SI groups (all *p* > 0.05). However, significant differences between the two groups were observed in almost all clinical variables. Specifically, the SI group had significantly higher HB traits (*p* < 0.001), HB symptoms (*p* < 0.001), employee-focused emotional labor (*p* < 0.001), job-focused emotional labor (*p* = 0.001), depression (*p* < 0.001), anxiety (*p* < 0.001), perceived stress (*p* < 0.001), trait anger (*p* < 0.001), state anger (*p* < 0.001), anger-in (*p* < 0.001), and anger-out (*p* < 0.001). Furthermore, the SI group had significantly more subjective health status responses of “bad” or “very bad” (*p* = 0.004). However, no significant differences were observed between the anger-control groups (*p* = 0.064) (Table 1).

**Table 1:** Baseline characteristics of the participants.

Variables		Total (*n* = 457)	Non_SI (*n* = 283)	SI (*n* = 174)	X^2^ or t	*P*-value
Sex	Men	243	140	103	4.093	0.053
	Women	214	143	71		
Age	19–29	126	80	76	0.918	0.632
	30–39	152	97	55		
	40–44	179	106	73		
	Raw (year)	34.75 ± 7.45	34.35 ± 7.58	35.40 ± 7.20	−1.460	0.145
Generation	M generation	267	157	110	2.658	0.118
	Z generation	190	126	64		
Marriage	Unmarried or divorced	281	174	107	0.000	1.000
	Married	176	109	67		
Residence area	Metropolitan	271	165	106	0.305	0.624
	Other	186	118	68		
Subjective health status	Good or very good	157	109	48	11.155	0.004^**^
	Normal	225	139	86		
	Bad or very bad	75	35	40		
Chronic disease	Presence	333	210	123	0.673	0.449
	Absence	124	73	51		
HB scale	HB traits	33.12 ± 9.16	31.01 ± 8.62	36.55 ± 8.99	−6.554	0.000***
	HB symptoms	25.85 ± 11.65	20.90 ± 9.76	33.91 ± 9.84	−13.799	0.000^***^
Employee-focused EL	Subtotal	3.27 ± 0.65	3.17 ± 0.66	3.54 ± 0.71	−3.586	0.000^***^
	Superficial acting	3.38 ± 0.76	3.27 ± 0.78	3.54 ± 0.71	−3.244	0.001^**^
	Deep acting	3.16 ± 0.75	3.07 ± 0.78	3.03 ± 0.69	−2.873	0.004^**^
Job-focused EL	Subtotal	3.09 ± 0.69	3.00 ± 0.71	3.24 ± 0.64	−3.203	0.001^**^
	Frequency of interactions	3.22 ± 0.90	3.16 ± 0.97	3.29 ± 0.79	−1.293	0.197
	Duration of interactions	3.20 ± 0.85	3.13 ± 0.88	3.31 ± 0.78	−1.927	0.055
	Variety of expressions	2.90 ± 0.91	2.74 ± 0.93	3.14 ± 0.82	−4.048	0.000^***^
Total EL		3.17 ± 0.61	3.07 ± 0.62	3.32 ± 0.56	−3.763	0.000^***^
DASS-21	Depression	7.09 ± 5.32	4.34 ± 3.94	11.56 ± 4.09	−18.729	0.000^***^
	Anxiety	5.70 ± 4.95	3.28 ± 3.48	9.64 ± 4.42	−17.065	0.000^***^
	Stress	8.26 ± 4.87	6.15 ± 4.14	11.68 ± 3.93	−14.119	0.000^***^
STAXI	State anger	16.71 ± 6.86	13.53 ± 4.61	21.90 ± 6.76	−15.720	0.000^***^
	Trait anger	19.74 ± 6.48	17.61 ± 5.46	23.19 ± 6.54	−9.815	0.000^***^
	Anger-control	19.36 ± 4.18	19.08 ± 4.34	19.82 ± 3.86	−1.854	0.064
	Anger-out	14.79 ± 4.68	13.05 ± 3.63	17.61 ± 4.82	−11.496	0.000^***^
	Anger-in	17.52 ± 5.00	15.98 ± 4.58	20.02 ± 4.64	−9.123	0.000^***^

DASS-21, the Depression, Anxiety, and Stress Scale-21; EL, emotional labor; HB, hwa-byung; SI, suicidal ideation; STAXI, The State–Trait Anger Expression Inventory. ^*^, *p* < 0.05; ^**^, *p* < 0.01; ^***^, *p* < 0.001.

### 3.2 Factors associated with the presence of SI

Multivariable logistic regression analysis was conducted with all variables simultaneously entered into the model. After mutual adjustment for all variables, factors significantly associated with the presence of “a little” or more SI were HB symptoms (aOR, 1.05; 95% CI, 1.00–1.11; *p* = 0.050), depression (aOR, 1.41; 95% CI, 1.23–1.62; *p* < 0.001), and state anger (aOR, 1.14; 95% CI, 1.05–1.24; *p* = 0.002). Factors significantly associated with the presence of “much” or more SI were age (aOR, 1.11; 95% CI, 1.02 to 1.21; *p* = 0.013), HB symptoms (aOR, 1.08; 95% CI, 1.01–1.16; *p* = 0.031), depression (aOR, 1.70; 95% CI, 1.40–2.06; *p* < 0.001), and state anger (aOR, 1.14; 95% CI, 1.02–1.27; *p* = 0.021). Finally, the only factor significantly associated with the presence of “very much” SI was depression (aOR, 1.65; 95% CI, 1.15–2.37; *p* = 0.006) (Table 2).

**Table 2:** Factors associated with the presence of suicidal ideation from multivariable logistic regression analysis.

Variables		Any SI		Much or more SI		Very much SI	
		OR (95% CI)	*P*-value	OR (95% CI)	*P*-value	OR (95% CI)	*P*-value
Sex (ref. Women)	Men	1.26 (0.61, 2.62)	0.527	0.96 (0.39, 2.37)	0.934	0.80 (0.19, 3.43)	0.766
Age		1.02 (0.95, 1.09)	0.601	1.11 (1.02, 1.21)	0.013^*^	1.01 (0.88, 1.14)	0.933
Marriage (ref. married)	Unmarried or divorced	1.42 (0.63, 3.19)	0.395	1.71 (0.62, 4.73)	0.300	1.99 (0.36, 10.92)	0.431
Residence area (ref. Metropolitan)	Other	0.91 (0.46, 1.82)	0.795	0.93 (0.37, 2.32)	0.871	1.84 (0.49, 6.92)	0.368
Subjective health status (ref. bad)	Good or very good	3.30 (0.97, 11.24)	0.056	1.88 (0.48,7.44)	0.369	1.12 (0.13, 9.71)	0.917
	Normal	0.93 (0.34, 2.57)	0.895	1.04 (0.33, 3.29)	0.941	0.80 (0.12, 5.30)	0.816
Chronic disease (ref. absence)	Presence	1.15 (0.53, 2.50)	0.732	0.46 (0.16, 1.28)	0.138	0.78 (0.17, 3.63)	0.748
HB scale	HB traits	0.99 (0.93, 1.05)	0.711	0.95 (0.87, 1.03)	0.202	0.95 (0.84, 1.07)	0.412
	HB symptoms	1.05 (1.00, 1.11)	0.050^*^	1.08 (1.01, 1.16)	0.031^*^	1.12 (0.98, 1.28)	0.097
EL	Employee-focused EL	0.57 (0.25, 1.30)	0.179	1.65 (0.49, 5.59)	0.423	0.79 (0.13, 5.03)	0.806
	Job-focused EL	1.06 (0.54, 2.08)	0.867	0.93 (0.39, 2.25)	0.880	1.35 (0.39, 4.59)	0.636
DASS-21	Depression	1.41 (1.23, 1.62)	0.000^***^	1.70 (1.40, 2.06)	0.000^***^	1.65 (1.15, 2.37)	0.006^**^
	Anxiety	1.09 (0.96, 1.24)	0.176	0.98 (0.84, 1.14)	0.755	0.89 (0.71, 1.11)	0.296
	Stress	0.90 (0.77, 1.06)	0.219	0.82 (0.65, 1.03)	0.093	0.89 (0.63, 1.26)	0.517
STAXI	State anger	1.14 (1.05, 1.24)	0.002^**^	1.14 (1.02, 1.27)	0.021^*^	0.93 (0.79, 1.09)	0.384
	Trait anger	0.99 (0.91, 1.07)	0.801	0.97 (0.88, 1.09)	0.644	1.03 (0.87, 1.23)	0.733
	Anger-control	1.03 (0.93, 1.14)	0.543	0.99 (0.87, 1.13)	0.883	0.97 (0.80, 1.18)	0.777
	Anger-out	1.04 (0.93, 1.17)	0.486	1.09 (0.95, 1.25)	0.236	1.23 (0.95, 1.59)	0.119
	Anger-in	0.94 (0.85, 1.05)	0.259	0.98 (0.87, 1.11)	0.787	0.98 (0.80, 1.20)	0.842

CI, confidence interval; DASS-21, the Depression, Anxiety, and Stress Scale-21; EL, emotional labor; HB, hwa-byung; OR, odds ratio; SI, suicidal ideation; STAXI, The State–Trait Anger Expression Inventory. All odds ratios are mutually adjusted for all other variables in the model. ^*^, *p* < 0.05; ^**^, *p* < 0.01; ^***^, *p* < 0.001.

### 3.3 Mediation analysis

**Figure 2:**
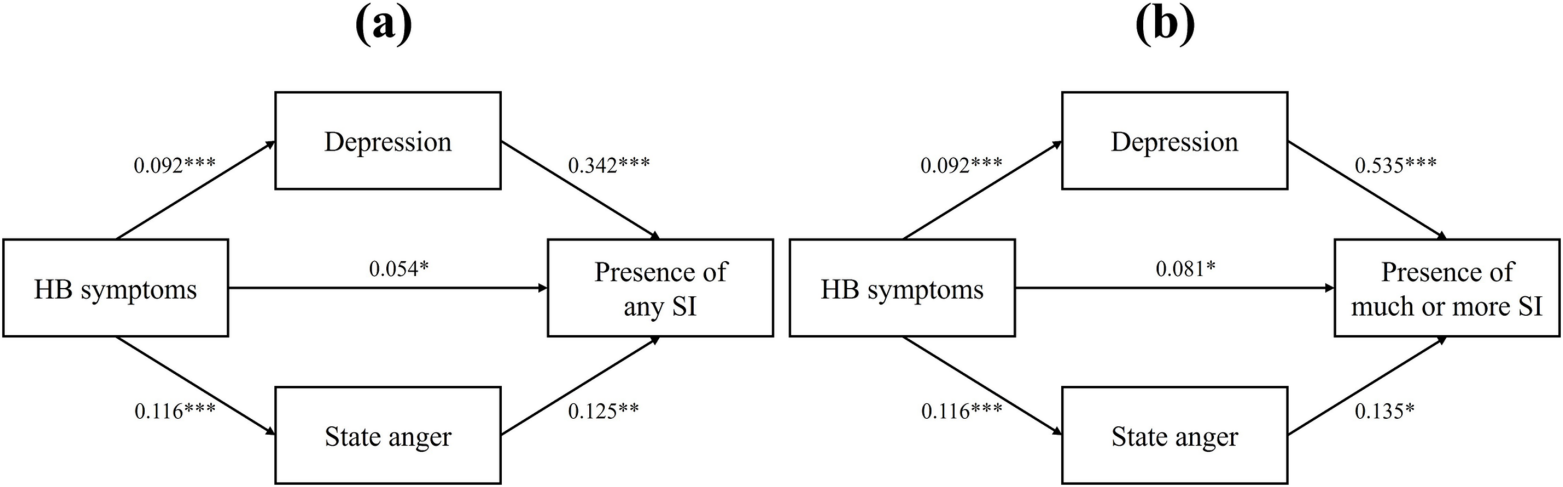
Mediation analysis examining whether HB symptoms predict the presence of **(A)** any SI or **(B)** much or more SI. *p < 0.05, **p < 0.01, ***p < 0.001.

A mediation analysis was conducted to examine the potential mediating roles of depression and state anger in the relationship between HB symptoms and the presence of “a little” or more SI. The results indicated that HB symptoms had a significant direct effect on SI (*β* = 0.054; 95% CI, 0.001–0.107; *p* = 0.045). Significant indirect effects were also observed through depression (*β* = 0.032, 95% CI, 0.015–0.063) and state anger (*β* = 0.015, 95% CI, 0.003–0.037). The total indirect effect was also significant (*β* = 0.046, 95% CI, 0.027–0.087) (Figure 2A; Table 3). Similar mediating effect of depression (*β* = 0.049, 95% CI, 0.028–0.109) and state anger (*β* = 0.016, 95% CI, 0.001–0.048) was observed in the relationship between HB symptoms and the presence of “much” or more SI. In this case as well, HB symptoms were significantly and directly related to the presence of SI (*β* = 0.081; 95% CI, 0.009–0.153; *p* = 0.028). These results suggest that the relationship between HB symptoms and SI is partially mediated by both depression and state anger (Figure 2B; Table 3).

**Table 3:** Mediation analysis of depression and state anger on hwa-byung symptoms-suicidal ideation relationship.

Variables		Any SI				Much or more SI			
		β (95% CI)	SE	Z or t-value	P-value	β (95% CI)	SE	Z or t-value	*P*-value
Direct	HB symptoms → SI	0.054 (0.001, 0.107)	0.027	2.007	0.045^*^	0.081 (0.009, 0.153)	0.037	2.198	0.028^*^
Indirect	Total	0.046 (0.027, 0.088)	0.015	–	–	0.065 (0.040, 0.139)	0.026	–	–
	via Depression	0.032 (0.015, 0.064)	0.012	–	–	0.049 (0.027, 0.109)	0.021	–	–
	via State anger	0.015 (0.003, 0.038)	0.009	–	–	0.016 (0.001, 0.049)	0.012	–	–
Paths	HB symptoms → depression	0.092 (0.051, 0.134)	0.021	4.363	0.000^***^	0.092 (0.051, 0.134)	0.021	4.363	0.000^***^
	HB symptoms → state anger	0.116 (0.049, 0.184)	0.034	3.412	0.000^***^	0.116 (0.049, 0.184)	0.034	3.412	0.000^***^
	Depression → SI	0.342 (0.204, 0.480)	0.070	4.854	0.000^***^	0.055 (0.341, 0.729)	0.099	5.402	0.000^***^
	State anger → SI	0.125 (0.044, 0.206)	0.041	3.034	0.002^**^	0.135 (0.024, 0.245)	0.057	2.384	0.017^*^

CI, confidence interval; HB, hwa-byung; SI, suicidal ideation; SE, standard error. ^*^, *p* < 0.05; ^**^, *p* < 0.01; ^***^, *p* < 0.001. Sex, age, marriage, residence area, subjective health status, presence of chronic disease, HB traits, employee-focused emotional labor, job-focused emotional labor, anxiety, stress, trait anger, anger-control, anger-out, and anger-in were included in the analysis as covariates variables. This analysis was based on 5,000 bootstrap samples.

### 3.4 Correlation between HB symptoms and the severity of SI

Pearson’s correlation coefficient between each question assessing HB symptoms and SI severity was calculated. The correlation coefficients ranged from 0.241 to 0.536. The questions with the highest correlations were in the following order: Q7 (coefficient = 0.536), Q1 (coefficient = 0.504), Q3 (coefficient = 0.489), Q5 (coefficient = 0.480), and Q10 (coefficient = 0.476) (Table 4).

**Table 4:** Correlation between hwa-byung symptoms and the severity of suicidal ideation.

SI Correlation with SI severity		HB symptoms in the HB scale (Q1 to Q15)	Dimensions of each symptom (physical, emotional, or cognitive)
Pearson correlation	*P*-value		
0.536	0.000	Q7. My hands and feet tremble, and I feel restless.	Physical and Emotional
0.504	0.000	Q1. My life is rather unhappy.	Cognitive
0.489	0.000	Q3. I feel that my life is sorrowful.	Emotional
0.480	0.000	Q5. I feel wronged.	Emotional
0.476	0.000	Q10. I often feel a heat buildup in my chest.	Physical
0.464	0.000	Q4. I feel sorrowful.	Emotional
0.458	0.000	Q6. My nerves are so fragile that I cannot control my emotions.	Emotional
0.430	0.000	Q11. I often feel something rising from below (legs or abdomen) to above (chest).	Physical
0.426	0.000	Q9. My face often flushes with heat.	Physical
0.373	0.000	Q8. I often feel disappointed in myself.	Cognitive
0.372	0.000	Q13. I have indigestion and often feel bloated.	Physical
0.360	0.000	Q12. When I get angry, my hands feel numb or tremble.	Physical
0.347	0.000	Q15. I feel that the world is unfair.	Cognitive
0.337	0.000	Q2. There are times when I feel deep regret or resentment.	Emotional and Cognitive
0.241	0.000	Q14. I am extremely tired.	Physical

HB, hwa-byung; SI, suicidal ideation.

## 4 Discussion

South Korea’s high suicide rate presents an urgent mental health crisis for the country (OECD, 2024). Suicide prevention policies in this country have continued to improve, but attention has been mainly paid to depression (Park, 2023), with relatively little consideration of the impact of anger. However, given the sensitivity of generation MZ to unfairness (Hong and Hong, 2023; Moon et al., 2024) and the presence of HB associated with Korean culture (Lee et al., 2014), the association between anger and the presence of HB and SI in this generation should be investigated.

In this study, the prevalence of SI was 38.07%. This high prevalence appears to be due to the fact that the existence of weak SI is included, and if limited to the prevalence of “very much” SI, it is 4.81%, which is similar to the prevalence of SI in the general population, found in other studies (i.e., 3.7 to 5.4%) (Hwang and Park, 2021; The Korean Society Of Oriental Neuropsychiatry, 2021; Kim E. Y. et al., 2023). However, passive or weak SI, which constitutes the SI spectrum, is also closely related to suicide attempts and is important from a mental health perspective (Liu et al., 2020; Harmer et al., 2024). Despite excluding individuals diagnosed with mood disorders, the high prevalence of SI in this study suggests that the Korean population with MZ may be exposed to high rates of mental health problems. While the coronavirus disease 2019 pandemic has had a more negative impact on mental health, including suicide risk, among younger generations in South Korea (Park et al., 2022), several other factors may contribute to this high prevalence. The MZ generation in South Korea faces unique socioeconomic challenges including severe housing affordability issues, high youth unemployment rates, gender inequalities and extreme competition in education and employment (Hwang and Shin, 2023; Kang et al., 2023). Additionally, this generation experiences significant economic inequality and limited social mobility compared to previous generations, often described as the “N-po generation” (N-giving-up generation) who have given up on various life goals such as marriage, home ownership, and having children due to economic constraints (Seo, 2019). The perceived generational inequity and social pressure to succeed despite limited opportunities may contribute to psychological distress in this population (Moon and Kim, 2023). Furthermore, Korea’s intensive work culture, characterized by long working hours and hierarchical relationships, may particularly affect the MZ generation who value work-life balance and fair treatment (Hong and Hong, 2023).

In the comparison between the SI and non-SI groups, the SI group showed poor overall mental health; however, it showed low scores on deep acting, a type of emotional labor that modifies inner feelings (Grandey, 2003). This result is consistent with existing studies showing that deep acting is associated with better mental outcomes in workers exposed to emotional labor (Grandey, 2003; Zhao et al., 2020). However, there is still a lack of research that divides emotional labor into surface acting and deep acting and investigates their relationship with suicidality. Considering that emotional labor has a serious impact on workers’ physical and mental health (Hwang et al., 2020), it appears that more in-depth research is needed in the context of suicidality. The relatively weak relationship between emotional labor and SI in our sample might reflect the heterogeneous occupational composition of participants, as emotional labor demands vary significantly across different job sectors. Additionally, recent workplace policy changes in South Korea regarding emotional labor protection might have helped mitigate its negative mental health impacts. Furthermore, for the MZ generation, other psychosocial stressors might play more prominent roles in SI risk than work-related emotional demands.

The relatively low mean scores on the emotional labor scale, despite high levels of unexpressed anger and perceived injustice, suggest that the sources of anger in this population may extend beyond workplace emotional demands. The MZ generation’s experience of anger and injustice appears to stem from broader societal issues, including significant economic disparities between generations, limited social mobility, systemic barriers to achieving traditional life goals such as home ownership and marriage, and gender inequalities (Hwang and Shin, 2023; Kang et al., 2023). This generation has been particularly affected by rapidly increasing housing prices, stagnant wages, and intense competition for stable employment, leading to a sense of relative deprivation compared to previous generations (Seo, 2019). Furthermore, their heightened sensitivity to fairness and justice (Moon and Kim, 2023) may make them more susceptible to perceiving and responding to various forms of social inequity beyond the workplace context. These findings suggest that interventions aimed at addressing anger and suicide risk in this population should consider not only workplace factors but also broader societal and generational issues contributing to perceived injustice.

Depression and state anger were significantly related to the presence of SI in the study group of interest (Masood et al., 2018; Orsolini et al., 2020). An important finding of this study was the confirmation of a significant association between SI and HB symptoms. The mediation analysis provided further insight into the relationship between HB symptoms and SI. While HB symptoms showed a direct effect on SI, they also exhibited indirect effects through depression and state anger. This suggests that HB symptoms may increase the risk of SI not only directly but also by exacerbating depression and state anger. These findings highlight the complex interplay between culture-bound syndromes, emotions, and SI. They also underscore the importance of addressing both HB symptoms and associated psychological factors in suicide prevention strategies for the Korean MZ generation.

HB is not a mental disorder that simply displays feelings of anger, but is also accompanied by characteristic physical symptoms caused by the accumulation of anger caused by injustice that cannot be expressed (Lee et al., 2014; Kwon et al., 2020). In Korean medicine, HB is caused by suppressed anger, which is likened to fire; therefore, its characteristic physical symptoms include heat sensation (Q9 and 10) or pushing-up in the chest (Q11) (Kwon et al., 2020). According to the results of the correlation analysis conducted in this study, these characteristic physical symptoms are highly correlated with the severity of SI (coefficient = 0.426 to 0.476). These were identified as unique symptoms of HB that were not significantly related to depressed mood (Min et al., 2009).

Importantly, in some cases, physical symptoms mask the presence of SI. SI and depression are highly prevalent among patients visiting primary care facilities in South Korea; however, they are likely to be underdiagnosed (Choi and Lee, 2017). Clinicians should identify risks early through appropriate interviews, even if patients do not spontaneously complain of SI (Choi and Lee, 2017). To achieve this, it is important to identify the various mental and physical signs and symptoms associated with the presence of SI. The significant association of HB symptoms with the presence of SI in Korea’s generation MZ found in this study can be considered helpful in identifying unidentified patients with SI in the future. In particular, Korean medicine doctors have not been included in South Korea’s mental health policy (Kwon and Lee, 2024). Considering that HB is a mental disorder that originated in Korean medicine and is generally treated by Korean medicine doctors in this country (Kwon et al., 2020), the role of Korean medicine doctors in preventing and managing suicide in patients with HB should be emphasized.

A significant association between physical and mental multimorbidities and suicidality has been reported (Renemane et al., 2021). HB includes unique somatic and behavioral symptoms related to the release and suppression of anger (Min, 2008; Kavalidou et al., 2019). It is also considered a functional somatic syndrome (Kwon et al., 2020). Therefore, HB can be considered a mental disorder with the characteristics of physical-mental multimorbidity. However, HB is a culture-related syndrome (Kwon et al., 2020) and is mainly diagnosed in South Korea; therefore, it can be considered a physical-mental multimorbidity in other countries. In the future, investigating the prevalence of HB as a type of physical-mental multimorbidity and its relationship with suicidality in populations in other countries may be proposed as a meaningful research topic.

The limitations of this study were as follows. First, the survey was distributed to a panel of survey companies; therefore, it is difficult to view it as a nationally representative sample. Therefore, the results of this study are challenged by their generalizability and may be influenced by surveys with larger or nationally representative samples. Furthermore, because HB is considered a syndrome related to Korean culture (Lee et al., 2014), there are limitations in applying these results to populations outside of Korea. Second, although web-based surveys are convenient, they may have methodological weaknesses, such as selective participation (Heiervang and Goodman, 2011). This means that the survey may have selectively attracted participants who were interested or had problems related to mental health, which may be related to the high prevalence of SI found in this study. Third, suicidality is a phenomenon accompanied by complex pathophysiological factors (Orsolini et al., 2020) and qualitative information, such as interpersonal experiences and perceptions may be involved (McClelland et al., 2022), but due to the limitations of the online survey, they were not investigated in this study. However, in the clinical setting, individual narratives should be considered when assessing suicidality. Fourth, while we focused on emotional labor as a work-related factor, we acknowledge that other important risk factors for SI, such as substance use, family history of suicide, and impulsivity, were not assessed in this study. Future research should consider incorporating these variables to provide a more comprehensive understanding of suicide risk in this population. Fifth, another important limitation is that we only assessed emotional labor among various work-related variables. Other occupational factors such as job position, working hours, work-life balance, financial compensation, and general working conditions could significantly influence both emotional labor demands and mental health outcomes (Kim, 2023). Furthermore, the impact of emotional labor likely varies across different occupations and job sectors. For instance, workers in customer service (Totterdell and Holman, 2003), healthcare (Chen et al., 2022), or education (Zheng et al., 2024) might experience higher emotional labor demands compared to those in other sectors. Future studies should consider a more comprehensive assessment of work-related variables and potentially conduct occupation-specific analyses to better understand the relationship between work conditions, HB symptoms, and SI in the MZ generation. Sixth, a major limitation of this study is the use of a single item to assess SI, rather than validated multi-item scales such as the Modified Scale for Suicidal Ideation, the Suicidal Ideation Attributes Scale, or the Beck Scale for Suicide Ideation. These validated scales would have provided more nuanced assessment of SI severity and characteristics, potentially revealing more detailed relationships between HB symptoms and specific aspects of SI. Future studies should employ comprehensive suicide risk assessment tools to better understand the relationship between HB and SI. Finally, we note that our analytical approach to SI - using it as both a dichotomous and ordinal variable - while serving different analytical purposes, may have some limitations. Future studies might benefit from consistent treatment of SI as an ordinal variable using methods such as ordinal logistic regression, which could provide additional insights while maintaining the ordered nature of the variable.

## 5 Conclusion

This study is the first to find results suggesting that the severity of HB symptoms is a risk factor for SI in the MZ population in South Korea, both directly and indirectly through its effects on depression and state anger. Given the high suicide rate in the country and the need to improve related mental health policies, the findings of this study can be used to improve future suicide prevention policies. Furthermore, this study highlights the role of medical personnel in KM clinics, where HB is diagnosed primarily, in the context of suicide prevention. However, because this study has limitations in generalizability, the results need to be confirmed using a larger or nationally representative sample in the future.

## Data Availability

The raw data supporting the conclusions of this article will be made available by the author, without undue reservation.
